# The Efficient Energy Collection of an Autoregulatory Driving Arm Harvester in a Breeze Environment

**DOI:** 10.3390/mi14112032

**Published:** 2023-10-31

**Authors:** Chao Zhang, Xinlong Yang, Boren Zhang, Kangqi Fan, Zhiming Liu, Zejia Liu

**Affiliations:** School of Mechano-Electronic Engineering, Xidian University, Xi’an 710071, China; 22041212928@stu.xidian.edu.cn (X.Y.); 21041212037@stu.xidian.edu.cn (B.Z.); kangqifan@gmail.com (K.F.); 23041212625@stu.xidian.edu.cn (Z.L.); 23041212878@stu.xidian.edu.cn (Z.L.)

**Keywords:** autoregulatory driving arm, low start-up speed, self-adjustable rotational inertia, constant driving arm

## Abstract

Breezes are a common source of renewable energy in the natural world. However, effectively harnessing breeze energy is challenging with conventional wind generators. These generators have a relatively high start-up wind speed requirement due to their large and steady rotational inertia. This study puts forth the idea of an autoregulatory driving arm (ADA), utilizing a stretchable arm for every wind cup and an elastic thread to provide adjustable rotational inertia and a low start-up speed. The self-adjustable rotational inertia of the harvester is achieved through coordinated interaction between the centrifugal and elastic forces. As the wind speed varies, the arm length of the wind cup automatically adjusts, thereby altering the rotational inertia of the harvester. This self-adjustment mechanism allows the harvester to optimize its performance and adapt to different wind conditions. By implementing the suggested ADA harvester, a low start-up speed of 1 m/s is achieved due to the small rotational inertia in its idle state. With the escalation of wind speed, the amplified centrifugal force leads to the elongation of the driving arms. When compared to a comparable harvester with a constant driving arm (CDA), the ADA harvester can generate more power thanks to this stretching effect. Additionally, the ADA harvester can operate for a longer time than the CDA harvester even after the wind has stopped. This extended operation time enables the ADA harvester to serve as a renewable power source for sensors and other devices in natural breeze environments. By efficiently utilizing and storing energy, the ADA harvester ensures a continuous and reliable power supply in such settings.

## 1. Introduction

With the focus on sustainable development in contemporary society, the earth has gradually entered the intelligent stage, which is mainly represented by smart transportation and smart cities [1]; these require clean, renewable, and distributed energy to meet the enormous energy demand of the planet’s trillions of sensors [2,3,4,5]. Because of this, it is challenging for traditional energy supply methods to meet growing needs, and this results in new demands for energy delivery. For a very long time in the past, distributed sensors have relied on a power grid or on batteries for their power supply, and this has had some seriously detrimental implications for the environment [6,7,8,9,10]. Distributed energy requires the development of more sophisticated technology. Due to its extensive availability and lack of pollution, wind energy has significant potential to resolve the issues listed above [11,12,13,14,15,16,17,18].

Low utilization rates and significant energy loss are two issues that low-speed wind energy gathering must still confront [19,20,21]. It is noteworthy that there are significant global reserves of breeze energy, and rotational energy harvesters are capable of effectively linking and electrostatically inducing mechanical energy into electrical energy. A rotational energy harvester is therefore a good choice for distributed energy gathering. Currently, the mechanism, circuits, components, and theoretical foundation of rotational energy harvesters can be enhanced [22,23,24,25,26,27,28,29]. Driving torque is one of the key factors influencing how well a harvester performs, yet breeze energy has the characteristics of unpredictability, volatility, and instability [30,31,32,33]. A self-power supply device based on triboelectric nanogenerators and electromagnetic generators was presented by [34]. The electromagnetic triboelectric hybrid generator-based energy harvesting and sensing gadget can inadvertently monitor wind speed. However, this device obtains a significantly high start-up wind speed because of its large and continuous spinning inertia. The triboelectric/electromagnetic hybrid nanogenerator was created by [14] for self-powered sensors. However, because the harvester’s driving arm length is fixed, it is challenging to adjust to the shifting wind energy in the surrounding environment. Thus, the output power can be effectively enhanced if the harvester’s arm length is automatically controlled by the mechanical structure [4,35,36]. Additionally, as the wind speed steadily drops, both the decay period and the recovered energy constantly rise [37,38].

The autoregulatory driving arm (ADA) harvester is offered in this research as a means of capturing input breeze energy. First off, by adjusting its own arm length, the ADA harvester can autonomously vary the parameters that determine the generator’s ability to produce power based on changes in wind speed. Therefore, the ADA harvester can dynamically reduce the gap between desired and actual power by adjusting its drive torque to match wind speed. However, the arm length of the corresponding constant driving arm (CAD) harvester is fixed and cannot be changed, so it cannot dynamically adjust its driving torque. This is the most essential difference between the two. In addition, ADA harvesters can achieve low start-up speeds, because they have a small rotational inertia in idle conditions. Furthermore, when the wind ceases, the ADA harvester can operate for a longer time compared to the CDA harvester. Due to these capabilities, The ADA harvester can efficiently use the remaining breeze energy and lengthen the time that harvested energy is retained. As a result, the ADA harvester demonstrates enhanced energy efficiency and prolonged energy storage capabilities. Experiments show that at 4.5 m/s of wind speed, the ADA harvester can generate 3.9 mW of power, which can reach 1.2 times the output power of the CDA harvester. After the wind stops at 4.5 m/s, the operating time of the ADA harvester is 10.9 s longer than that of the CDA harvester. Therefore, as a source of distributed energy, some sensors in the natural breeze environment can receive continuous electric energy from the ADA harvester.

## 2. Results and Discussion

### 2.1. Structure Design

**Figure 1:**
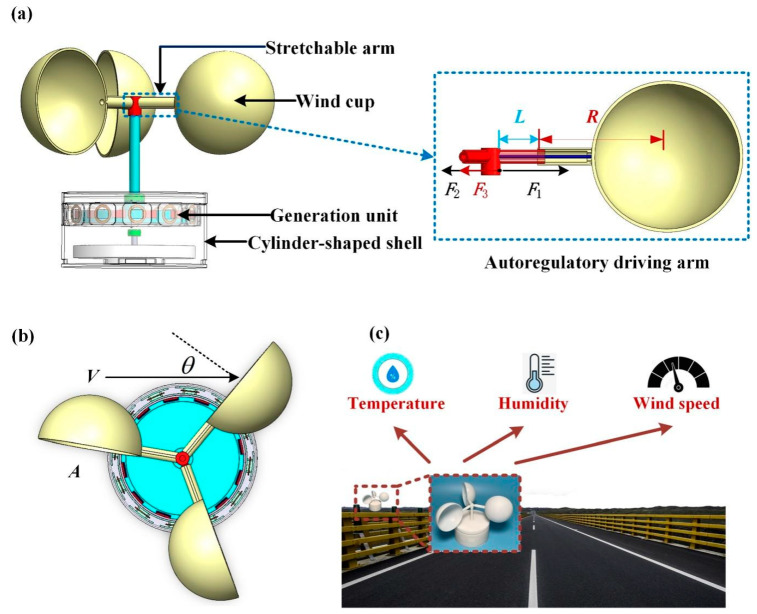
Diagram illustrating the structure of the ADA harvester. (**a**) Basic structure and autoregulatory driving arm unit. (**b**) Top view of the harvester. (**c**) Device configuration on the side of highway.

In Figure 1a, the structure of the ADA harvester is composed of three wind cups, three stretchable arms, a generation unit, and cylinder-shaped shell. Among them, each stretchable arm consists of a fixed end, a large tube, an elastic string, and a narrow arm. The ADA harvester can alter the arm length based on the coordinated functioning of the self-adjustment unit. The elastic string is used to allow the arm of the ADA harvester to adjust to a reasonable range in the breeze environment, which is shown in the autoregulatory driving arm unit *L*—the amount of elongation the elastic string experiences when the wind cup spins. *L* + *R* stands for the separation between the rotating axis and the wind cup’s center.

The ADA harvester is depicted in top view in Figure 1b, which usually consists of three hemispherical or parabolic conical hollow cups, and its wind cup is fixed on a three-pronged bracket at 120 degrees to each other. The concave surface of the cup shell is oriented in one direction, and the entire cross arm is locked on a vertical rotating axis. The wind cup rotates thanks to the dynamic torque as a result of the approaching breeze. In Figure 1b, *A* is the wind cup’s typically circular cross-sectional area; *θ* is the angle between actual wind speed *V* and the center line of the wind cup section. According to the definition of dynamic pressure, as the wind cup is stationary, the speed of the breeze relative to the wind cup is *V*. The photograph of the ADA harvester device in its as-built state is shown in Figure 1c. This device uses wind energy to power various sensors that monitor the environment, including wind speed, temperature, and humidity.

### 2.2. Theoretical Analysis

On the wind sensor’s rotational axis, there is friction. The minimum airflow speed necessary to cause the wind cup to precisely overcome the friction force and transition from static to continuous rotation is known as the start-up wind speed. When using a cup-type wind sensor, the torque generated by the wind pressure of the wind cup can be calculated in Equation (1).

(1) $$M=2Nu^{2}-Dun$$

where *Dun* is the air resistance torque and 2*Nu*^2^ is the torsional torque.

The resultant moment on the wind cup is 0 when the wind is steadily blowing. Currently, Equation (2) denotes the sum of the static friction torque *B*_0_ of the elastic rope and the elastic torque *B*_1_*n* of the string, and the combined torque *M* is exactly representative of the external force.

(2) $$B_{1}n+B_{0}=2Nu^{2}-Dun$$

When the wind cup is at rest, Equation (2) can be represented by Equation (3):

(3) $$u_{\min}=\sqrt{\frac{B_{0}}{2N}}$$

The start-up wind speed is referred to as *u*_min_. *N* is a constant calculated from 2*N* = *ρARa_m_*. Where *ρ* is the air density, *a_m_* is the average pressure coefficient, *A* is the wind cup’s incision area, and *R* is the wind cup’s rotational radius. The computation of the starting torque is realized using the start-up wind speed measurement in Equation (3).

When the rotational speed is 0, the start-up torque of the ADA harvester is 20 mm, smaller than that of the CDA harvester, whose start-up torque is 40 mm. Therefore, the start-up wind speed of the ADA harvester is lower than that of the CDA harvester in the breeze environment.

In Figure 1a, The ADA harvester exerts more force when the wind speed steadily increases. The elastic string force can be thought of as *F*_2_ = *kL*, where *k* is the string’s elastic modulus, and *L* is the Hooke’s theorem’s deformation variable. The opposing resultant force between the static friction force *F*_3_ and the elastic string force *F*_2_ is the centrifugal force *F*_1_. The only factor affecting the value of *F*_1_ is *F*_2_, while *F*_3_ is constant. Equation (4) shows the force balance that the rotating elastic string creates.

(4) $$\frac{1}{2}\rho V^{2}AC(\theta )=kL+F_{3}$$

Equation (5) shows the elastic deformation variable

(5) $$L=\frac{1}{2k}\rho V^{2}AC(\theta )-\frac{F_{3}}{k}$$

where *ρ* is 1.25 kg/m^3^, *A* is 3.848 × 10^−3^ m^2^, and the mean value of *C*(*θ*) is 1.5. Following this, an examination of how elastic strings with various diameters affect the value of *L* is undertaken.

### 2.3. Output Performance

**Figure 2:**
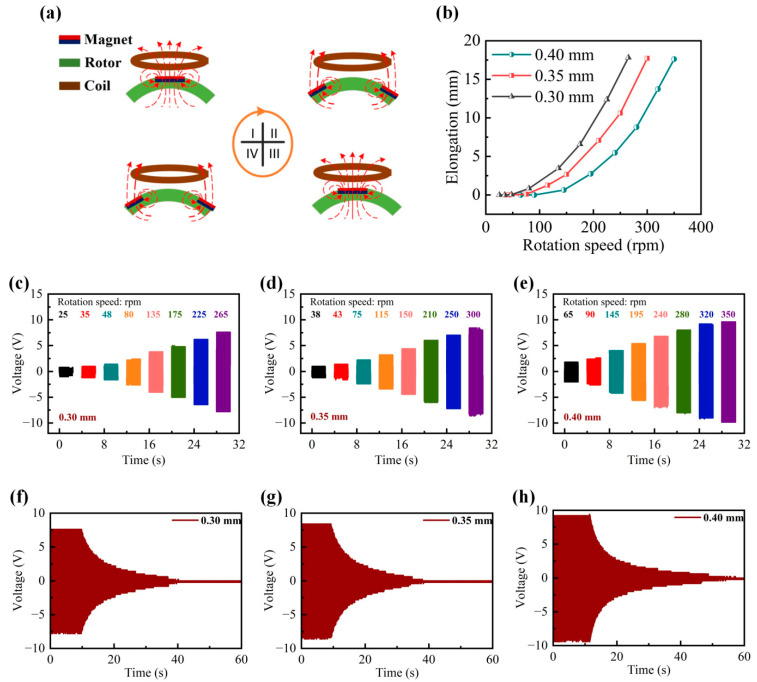
Harvester performance assessment. (**a**) Results of electromagnetic induction in different scenarios. (**b**) Elastic string elongation at various wind speeds (0.9 mm, 1.0 mm, and 1.2 mm diameters). (**c**) Open-circuit voltage with a 0.9 mm elastic string. (**d**) Open-circuit voltage with a 1.0 mm elastic string. (**e**) Open-circuit voltage with a 1.2 mm elastic string. (**f**) Open-circuit voltage after breeze energy removal with a 0.9 mm elastic string. (**g**) Open-circuit voltage after breeze energy removal with a 1.0 mm elastic string. (**h**) Open-circuit voltage after breeze energy removal with a 1.2 mm elastic string.

The electromagnetic induction phenomenon that the ADA harvester relies on is depicted in Figure 2a. The magnetic flux through the coil gradually increases as a magnet fixed to the rotor rotates toward it. The coil is where the induced current is produced. However, the succeeding magnetic field prevents the magnetic flux (I) from decreasing. The magnet moves away from the stationary coil as the rotor turns. The magnetic flux through the coil decreases as a result of this event, through an order to compensate for the lower magnetic flux (II); the current therefore flows through the coil in the opposite direction. The nearby magnet also deviates from the coil due to continual rotation, which results in the current flowing in alternate directions once more (III and IV). This causes the magnetic field to alternately shift directions while the rotor continuously rotates, which produces periodic alternating currents.

In Figure 2b, the range of rotation speeds for the three different elastic strings (diameter: 0.9, 1.0, and 1.2 mm) is 0.9–3.4, 1.2–3.6, and 1.5–4.5 m/s, respectively. It has been noted that the ADA harvester’s ability to withstand higher wind speeds improves with a greater stiffness of the elastic string. The output performance of the ADA harvester using various elastic strings is shown in Figure 2c–e. As the rotation speed rises, a trend of expansion in the open-circuit voltage is visible. However, the ADA harvester equipped with a 1.2 mm elastic string demonstrates higher open-circuit voltage output, as it can endure higher rotation speeds. Additionally, compared with the other two harvesters with elastic strings of 0.9 mm and 1.0 mm, it can work with a longer duration. In addition, the rotation speed of the ADA harvester with an elastic string of 1.2 mm can exactly match the wind speed range in the breeze environment. Thus, according to the common specification of elastic strings in the market, the 1.2 mm string was chosen for this study.

Equation (5) in Section 2.2 provides the theoretical range of *L*, which is displayed in Table 1. Within a certain range, *L* rises accordingly with the steady increase in wind speed.

**Figure 3:**
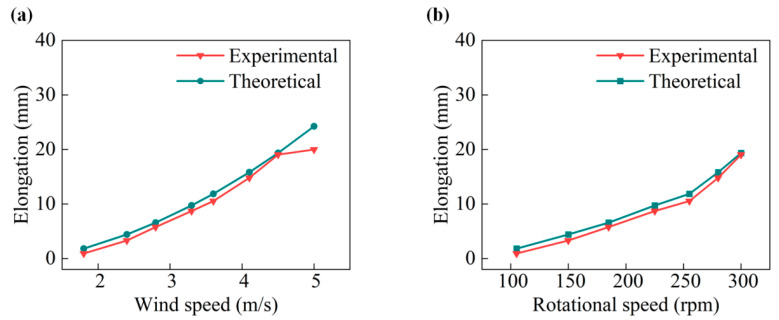
Analysis of the differences in length between theory and experiment in (**a**) various wind speeds and (**b**) various rotation speeds.

The value *L* of the elongation and related rotational speeds are carried out in various wind speeds to confirm the accuracy of the driving arm length theory for collecting breeze energy. Figure 3a,b shows the comparison between the theoretical and experimental elongation values, demonstrating a substantial agreement between the two.

It is worth noting that the self-adjustment range of elongation, denoted as *L*, is between 0 mm to 20 mm during the operational conditions of the equipment. This range corresponds to the wind speed increasing from 1.5 m/s to 4.5 m/s. As a result, the elongation *L* reaches its maximum length of 20 mm at speeds greater than 4.5 m/s.

## 3. Performance Comparison

**Figure 4:**
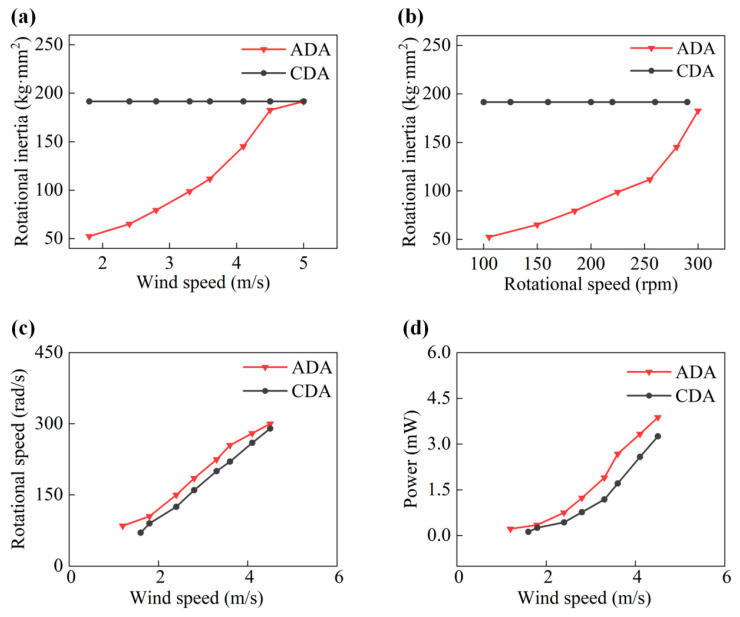
Performance of ADA and CDA harvesters: (**a**) Rotational inertia comparison at various wind speeds. (**b**) Rotational inertia comparison at various rotation speeds. (**c**) Rotational speed comparison at various wind speeds. (**d**) Output power comparison at various wind speeds.

In order to demonstrate that the ADA harvester achieves decreased start-up wind speed, rotational inertia at various wind speeds and related rotation speeds are contrasted in Figure 4a,b. The CDA harvester maintains steady and large rotational inertia throughout. By contrast, the ADA harvester exhibits smaller initial rotational inertia, indicating that its start-up torque is lower than that of the CDA harvester. Furthermore, the increasing wind speed results in an amplified centrifugal force, which leads to the extension of the driving arms in the ADA harvester, and the rotational inertia of the ADA harvester gradually rises.

In Figure 4c,d, comparing the CDA harvester and the ADA harvester at various speeds, the variation trend of output power and rotation speed are shown separately. The ADA harvester’s maximum arm length is 40 mm, its maximum peak output is 3.9 mW, and its maximum rotational speed is 300 rpm as the wind speed exceeds 4.5 m/s. Compared to the CDA harvester with a fixed arm length, the ADA harvester can automatically adjust its arm length as wind speed increases. In addition, ADA harvesters require lower starting air speeds than CAD harvesters, requiring only 1 m/s of wind speed to start.

**Figure 5:**
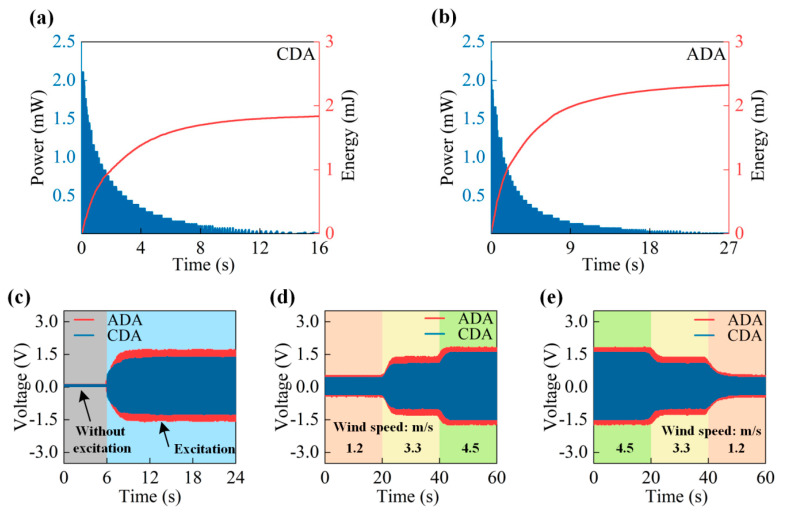
Comparison of ADA and CDA harvester performance: (**a**) CDA harvester power and energy generation after removing breeze energy. (**b**) ADA harvester power and energy generation after removing breeze energy. (**c**) Output voltage with wind speed increasing from 0 m/s to 4.5 m/s. (**d**) Output voltage with wind speed increasing from 1.2 m/s to 3.3 m/s to 4.5 m/s. (**e**) Output voltage with wind speed decreasing from 4.5 m/s to 3.3 m/s to 1.2 m/s.

Figure 5a,b shows the energy and power decay time for the two generators. The moment of the inertia of the ADA harvester gradually decreases with wind speed. When the wind is not very strong, the ADA harvester can continue to operate. However, because the moment of inertia cannot be changed, the CDA cannot operate when the wind speed is low. Therefore, after the wind completely ceases, ADA harvesters can work for longer. Similarly, while the power of the CDA harvester degrades to 0 mW in 15.5 s, the power of the ADA harvester degrades to 0 mW in 26.4 s, demonstrating the better energy retention of the ADA harvester. Additionally, the CDA harvester produces 1.9 mJ of energy as opposed to the ADA harvester’s 2.4 mJ of energy under identical conditions of reduced wind speed. Therefore, the CDA harvester is regarded as evidence of the ADA harvester’s efficacy. In Figure 5c, output voltage is compared between the two generators. The ADA harvester’s effective voltage during excitation is higher than that of the CDA harvester.

The comparative growing trend of voltage change under different wind speeds is presented in Figure 5d. The wind speed initially begins at 1.2 m/s and subsequently increases to 3.3 m/s after a 20 s interval. After an additional 20 s, the wind increases even further, reaching 4.5 m/s. As wind speed drops from 4.5 m/s to 1.2 m/s, a comparative downtrend of voltage change occurs at the same interval, as shown in Figure 5d. In particular, the ADA harvester can adjust the length of its arm on its own to dynamically match breeze energy to the generation unit. As a result, the wind cup can utilize the energy of the wind more effectively as wind speed changes.

**Figure 6:**
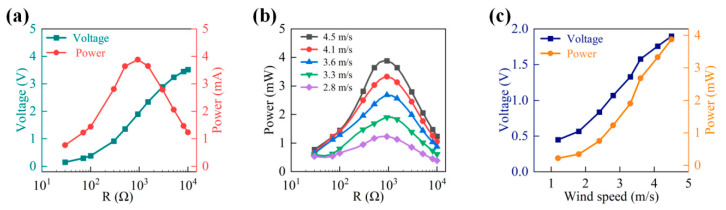
The performance test of the ADA harvester. (**a**) Output voltage and power under various loads. (**b**) Output power under various wind speeds and loads. (**c**) Output voltage and power under various wind speeds.

Figure 6a shows an examination of the impact of various load resistances on power outputs. Under a 4.5 m/s wind speed, generated voltage rises monotonously, with resistance ranging from 30 Ω to 10,000 Ω. In addition, the output power continues to rise, but it gradually dies down with a larger load resistance. When the load is 930 Ω, an output peak power of 3.9 mW can be achieved by the ADA harvester.

In Figure 6b, the output power of the ADA harvester achieves the maximum when the load resistance is about 930 Ω. In Figure 6c, as the wind speed rises from 1.2 to 4.5 m/s, the value of output power and voltage ascend from 0.22 mW/0.45 V to 3.9 mW/1.9 V, respectively. As a result, it is possible to demonstrate that the ADA harvester performs better at collecting wind energy.

## 4. Applications of the ADA Harvester

**Figure 7:**
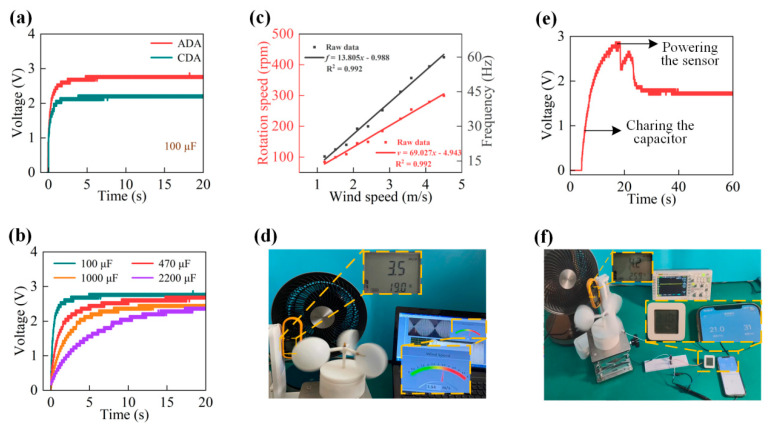
Uses for the ADA harvester. (**a**) Comparison of voltage curves stored by ADA and CDA harvesters for 100 μF capacitors (**b**) Voltage value curve of 100 µF, 47 µF, 1000 µF, and 2200 µF capacitors. (**c**) Rotor speed and output frequency of the ADA harvester. (**d**) A photo of a sensor that runs independently and is fueled by an ADA harvester. (**e**) Variation in voltage for a 2200 µF capacitor (**f**) A photo of an ADA harvester-powered wireless hygrothermograph.

The energy management unit employs a storage capacitor and a bridge rectifier to change the ADA harvester’s AC outputs to DC outputs, due to the randomness, volatility, and intermittency of wind energy. Additionally, the created energy can be stored in the capacitor for later use. In Figure 7a, the ADA and CDA harvesters charge the 100 F capacitor to 2.8 V and 2.24 V, respectively, under a wind speed of 4.1 m/s. When the wind speed is 4.1 m/s, as is shown in Figure 7b, various capacitors are used to charge the voltage trends. Within roughly 4.9 s, the 100 F capacitor is charged to 2.8 V. Additionally, a bigger 470 µF capacitor voltage can steadily increase until it reaches the saturated level of 2.7 V in just over 15 s. Accordingly, the ADA harvester’s higher charging capability is shown.

As the wind speed increases from 1.2 m/s to 4.5 m/s in Figure 7c, the rotor speed increases linearly from 85 rpm to 300 rpm. Similar outcomes are obtained when using the voltage signal, whose frequency increases linearly from 17 Hz to 60 Hz. Additionally, their linear relationship causes the ADA harvester to transform into a self-sufficient power source. Figure 7d shows the excellent alignment between the commercial anemometer and the wind speed sensor, which validates the promising potential of the ADA harvester.

Additionally, as shown in Figure 7e,f, a wireless hygrothermograph can gauge the temperature and humidity of the nearby area and send the data to a smartphone through Bluetooth, and it is powered by an ADA harvester. Additionally, a 2200 µF capacitor that serves as a reasonably reliable power supply for the sensors stores rectified electric energy. Additionally, at wind speeds of roughly 4.2 m/s, it takes the ADA harvester about 11 s to charge the capacitor to 2.8 V.

When the hygrothermograph is turned on, a rapid drop in the capacitor’s voltage is observed. This phenomenon happens because there is significant energy loss during system initialization and wireless connection formation. Additionally, after startup, the storage capacitor can gradually draw near to a 1.8 V steady voltage. As a result, the wireless hygrothermograph can be powered by the ADA harvester and continue operating continuously.

## 5. Conclusions

This paper presents the ADA harvester as a solution for achieving the adjustable correlation between the length of the generator’s driving arm and the incoming breeze energy. The ADA harvester is composed of a resilient string and a flexible arm for each wind cup. Since the rotational inertia can be adjusted, the ADA harvester enables a comparatively lower threshold for wind speed to initiate operation. Moreover, in situations where the wind speed is inconsistent, the ADA harvester can generate increased output power in natural breeze conditions. This is possible, because its driving torque dynamically adjusts to the wind speed on its own. Additionally, when the wind stops, a typical ADA harvester can operate for a longer time than a CDA harvester. The ADA harvester achieves synchronization with varying wind speeds by autonomously adjusting its driving torque. It outperforms the corresponding CDA harvester in several aspects. With a lower start-up wind speed requirement of 1 m/s, the ADA harvester can operate effectively. Its decay time of 26.4 s is significantly longer than that of the CDA harvester, allowing for a greater amount of energy storage during the same time span. As a result, the ADA harvester can be used as a reliable renewable energy source for self-powered electronics in breeze environments, delivering 3.9 mW of output peak power, which is 1.2 times more than the CDA harvester. The findings of this study can serve as valuable references for the design of harvesters.

## Figures and Tables

**Table 1:** Relationship between *V* and *L* based on theory.

*V*	*L*
1.8 m/s	1.8395 mm
2.4 m/s	4.4369 mm
2.8 m/s	6.5808 mm
3.3 m/s	9.72448 mm
3.6 m/s	11.85805 mm
4.1 m/s	15.82631 mm
4.5 m/s	19.37196 mm
5.0 m/s	24.26786 mm
